# Defining knowledge gaps in preterm birth research: Can biomarkers fill the gaps?

**DOI:** 10.3389/fmed.2025.1655833

**Published:** 2025-09-02

**Authors:** Scott M. Williams, Kevin P. Rosenblatt, Sandra Reznik, Dawn P. Misra, Shajila Siricilla, Nardhy Gomez-Lopez, Brandie D. Taylor, Kristina M. Adams Waldorf, Ramkumar Menon, PREBIC North America 2024, Addy Cecilia Helguera-Repetto

**Affiliations:** ^1^Cleveland Institute for Computational Biology, Case Western Reserve University, Cleveland, OH, United States; ^2^Department of Population and Quantitative Health Sciences, Case Western Reserve University School of Medicine, Cleveland, OH, United States; ^3^Brazos Neuroscience, Inc., Bellaire, TX, United States; ^4^NX Prenatal, Inc., Bellaire, TX, United States; ^5^Department of Pharmaceutical Sciences, St. John’s University, Queens, NY, United States; ^6^Department of Epidemiology and Biostatistics, Michigan State University, East Lansing, MI, United States; ^7^Department of Pediatric Neonatology, Vanderbilt University Medical Center, Nashville, TN, United States; ^8^Center for Reproductive Health Sciences, Departments of Obstetrics and Gynecology & Pathology and Immunology, Washington University School of Medicine, St. Louis, MS, United States; ^9^Advocate Aurora Research Institute, Milwaukee, WI, United States; ^10^Department of Obstetrics and Gynecology, University of Washington, Seattle, WA, United States; ^11^Department of Obstetrics and Gynecology, Division of Basic Science and Translational Research, The University of Texas Medical Branch at Galveston, Galveston, TX, United States

**Keywords:** pregnancy, prematurity, risk, inflammation, intervention, disparity

## Abstract

Preterm birth (PTB) is a syndrome arising from multiple etiologies that manifest as a final common phenotype, delivery before full term. Current knowledge gaps in epidemiologic, basic science, and clinical fields have limited our understanding of this complex pregnancy syndrome. Lack of insight into the cellular and molecular pathways underlying spontaneous PTB (PTB) has thus limited effective clinical management and restricted the investigation of novel treatments or druggable targets. Here, we examine several areas of domain-driven research that may lead to a better understanding of PTB, including infection and inflammation that drive early labor, social factors and their biological consequences that may affect or contribute to PTB risk, and current limitations affecting the development of novel pharmacological treatments. We discuss how the development of new biomarkers or panels of biomarkers can define PTB risk status and disease mechanisms and potentially lead to new therapies by bridging gaps between research domains often used to study PTB in relative isolation. We note that these panels may be population specific and it is critical to assess the heterogeneity of PTB in light of the variation among women of diverse backgrounds, both environmentally and genetically. Finally, we consider how complementary biomarkers from different PTB research domains could be integrated to design new diagnostic, preventative, and management options. Our hope is that new ways of looking at PTB can improve understanding of this common pregnancy complication leading to reduced global rates of PTB and improved outcomes for affected infants.

## Introduction

Preterm birth is a complex phenotype that includes multiple and independent etiologies and pathways that culminate in birth before 37 weeks gestation (1, 2). Diagnosing and managing PTB as a single disease is, therefore, clinically ineffective (1). Nonetheless, the typical approach of biomedical research into PTB focuses primarily on the single clinical outcome of gestational duration, thereby limiting our current understanding of the disease mechanisms and restricting novel perspectives (3, 4). Instead, preterm birth should be viewed as a syndrome that can primarily be divided broadly into indicated or iatrogenic, comprising a variety of spontaneous subtypes (1, 2). Iatrogenic preterm births occur in the context of medical indications to the mother or the fetus where specific conditions, including preeclampsia, gestational diabetes, and anomalies, may force early delivery (2, 5). In contrast, spontaneous preterm birth (referred to as PTB in the rest of this review), as the name indicates, develops without a known underlying etiology and progresses through various (or several) physiological pathways that culminate in early delivery (1, 2). PTB can be classified further as either preterm labor without rupture of the fetal membranes (amniochorion) or as preterm prelabor rupture of the membranes (PPROM) (6, 7). This subtyping may be very useful for defining specific biological pathways that help define druggable targets within the pathways, thereby benefiting from individualized interventions (8, 9). However, researchers examining PTB have tended to view the syndrome not through the likely underlying multiplicity of biological mechanisms but rather through a single lens defined by their training and backgrounds (10, 11). Hence, as has occurred with many other diseases, including some complex syndromes, there is a propensity for condensing PTB into preconceived bins based on clinical training and research background rather than new insights into biology. Consequently, treatments for PTB are less likely to be effective if the heterogeneity of etiologies is not explicitly addressed (12).

Dividing PTB into the current restrictive clinical domains and scientific silos is a natural outcome of the reductionist approach that has historically identified many disease mechanisms (13). However, multiple, complex phenomena can interact, so as to obscure, and mask each other’s effects on adverse pregnancy outcomes (14). Therefore, we propose a fresh approach without reductionist thinking to gain insights into PTB mechanisms. A step in this direction is to systematically define knowledge gaps in current research domains and examine how they interface with each other to better delineate the basic science areas most likely to translate into precision approaches to diagnose, manage, and prevent PTB. The knowledge gaps are in multiple realms, such as understanding the heterogeneity of basic etiological mechanisms and clinical presentations, and how they relate to possible interventions. Here, we define several of the knowledge domains relevant to PTB, some gaps within specific research silos, and, finally, we discuss how gaps between silos can be identified to define novel disease pathways better suited to interventions. This manuscript is not intended to be a comprehensive review of the prevailing clinical understanding of PTB or all knowledge gaps, but we aim to provide examples of how recognition of the gaps and filling them with appropriate reductionist approaches, when warranted, may lead to further insight and suggest new treatments and better patient management.

We examine several areas as examples of domain-driven research into PTB: (1) how infection and inflammation at the feto-maternal interface can perturb homeostatic balances, triggering quiescent uterine tissues into an active stage of labor; (2) how social factors may prompt biological changes that are causal of PTB; and, (3) how discovering pharmacological treatments is limited by our study designs and the limitations on working with pregnant women in clinical trials. Our perspective focuses on how biomarkers can connect signals of high-risk status for PTB across domains, define potentially distinct risk pathophysiologies, and help manage and prevent adverse outcomes. In this paper, we focus on the definition of biomarkers as described in the by the FDA and NIH described as BEST (Biomarkers, EndpointS, and other Tools) that defines and describes the development and use of biomarkers (15). We present a means to link the genetic factors and biomarkers associated with siloed domains and suggest how specific biomarkers may be used to infer novel treatment pathways, especially within the confines of immunological responses and social factors. Our goal is to not only address how to define markers of PTB within each silo but how we can use them as key links to design diagnostic, preventive, and management options to lower the prevalence and sequelae of PTB.

In our conceptualization, biomarkers fulfill many functions, serve as indicators of risk and exposures, and monitor the development and progression of pathologic events while still performing their traditional roles as diagnostic and prognostic indicators. The complexity of risk-driving factors has often hampered biomarker discovery in perinatal medicine. It has often been implicitly assumed that a single or a few biomarkers can be used to define most if not all, pregnancies at risk. However, the search for a limited number of markers and treatments under the assumptions of limited etiological heterogeneity is inherently flawed for a syndrome as complex as PTB.

## Infection and inflammation in PTB

Preterm birth is solely defined by gestational duration, but several pathologic processes can result in this outcome. Infection and inflammation in the amniotic cavity and placenta is a dominant factor associated with early PTB (2, 16–18). Intrauterine infection that occurs in the lower uterine segments and the intraamniotic cavity promotes myometrium contraction and cervical ripening thereby facilitating the process of delivery (2, 19–21). Hence, a major knowledge gap is how microbes traffic across the maternal-fetal interface to invade the amniotic fluid. The inflammatory response is propagated by host causing tissue injury that induces preterm labor. The development of diagnostics and therapeutics to reduce neonatal mortality and morbidity therefore requires a deeper understanding of the complexity of immunologic and infectious contributions (22–25).

Several infectious agents are associated with PTB, but the overall risk is limited to a rather small number of potentially pathogenic agents (24, 26–29). Furthermore, little is known about how microbial communities in the lower genital tract can modify the virulence potential of specific bacteria. For example, little is known about the microbial communities in the lower genital tract and specific associated bacterial virulence (30–34). Even less is known regarding non-bacterial promoters of immune responses and inflammation, such as viruses, although such associations exist (Box 1). In addition, immune crosstalk between maternal or fetal compartments and microbes may further affect risk, although this concept is also poorly understood (35, 36) Therefore, we need to better understand how exposure to a variety of individual infections and microbial communities induces PTB. In the same light, we need to know how an individual’s responses to infection vary and can lead to a preterm versus a term delivery. This latter point may be mediated by genetic predisposition or exogenous exposures before or during pregnancy that induce specific immune responses that can be causative.

BOX 1 Gaps in knowledge of infection and immunology in PTB

- There are unknown effects of highly diverse microbial communities in the lower genital tract and bacterial virulence factors on PTB.
- Several viral infections have been associated with a higher risk of PTB, but the mechanisms predisposing to PTB are unclear (108–114).
- The pathogenesis of sterile intra-amniotic inflammation is unknown in general.
- Placental-fetal immune crosstalk and its relationship with fetal health is poorly understood.
- Chronic placental inflammation is a poorly understood histopathologic diagnosis contributing to PTB.
- It is unknown how cytokine, metabolic, hormonal, nutritional, and environmental signals are integrated by placental immune cells to trigger PTB.
- Innate lymphoid cells (ILCs) are a rare immune cell type that responds to environmental signals and may play a key role at the maternal-fetal interface in immune tolerance and priming, pregnancy maintenance, and parturition timing.
- Investigation of the impact of specific cell death pathways on placental inflammation and PTB is limited but may represent critical triggers of PTB.
- The roles of fetal membrane inflammation and cervical Inflammation are poorly understood.
- Therapies related to infection and inflammation are ill-defined or unavailable but may be urgently needed (see Box 3).

Among cases of spontaneous preterm labor associated with infection and inflammation, one of the most common phenotypes is sterile intrauterine inflammation, which has been documented using amniocentesis (37, 38). However, the possibility that a large subset of PTB with sterile intrauterine inflammation is related to a resolved or localized bacterial infection in the choriodecidual space is intriguing (39–42). Infection and inflammation of the feto-maternal interface are difficult to study *in vivo* and in real-time but are often present after prolonged rupture of the fetal membranes or after the development of chorioamnionitis. Diagnosing such a condition therefore represents a major challenge to clinicians, as the timing of detection may be too late for effective intervention. Therefore, early detection of inflammation or diagnosis of immune-related risk, and continuous monitoring of immune status are critical to reducing the risk of many adverse pregnancies. Awareness of how we can effectively surveil these processes at appropriate times requires increased knowledge about immune-related biomarkers relevant to each category or subset of adverse outcomes.

## Social determinants of PTB

Multiple factors are associated with PTB, among them are social ones that are not defined by clearly delineated biological differences. Women in minority groups or from impoverished areas often experience a higher rate of PTB, especially those from impoverished countries across the world. Much of this disparity can be attributed to multiple social and structural factors (43, 44). In most cases, these disparities have persisted for decades with virtually no improvement (45). Throughout life, but particularly during pregnancy, experiences of racism and discrimination may cause severe trauma and stress, leading to negative health and pregnancy outcomes (43, 44, 46). Unfortunately, most clinical, prediction, and biomarker studies of PTB continue to use race/ethnicity as a proxy for genetic susceptibility, ignoring factors such as structural racism and other sociopolitical constructs that can impact reproductive and perinatal health independent of genetic ancestry. Of course, the overlap of social constructs with ancestry can make this a daunting task. The gaps in our understanding of the role of social determinants stem from several factors, such as an inability to develop or implement standardized measures of these determinants at multiple levels and across multiple contexts, from the individual to the society, encompassing structural and neighborhood characteristics (Box 2). There is also a gap in guidance for PTB researchers on how to model these variables and identify confounders that are common at each of these levels. It is also unclear how to analyze multiple exposure levels that can simultaneously affect PTB risk (1).

BOX 2 Gaps in knowledge of SDOH in PTB

- Standardized measures that can be implemented in preterm birth studies to quantify social inequality from the structural, neighborhood, and interpersonal levels are lacking in studies of PTB (115–122).
- There is still a need to develop and validate new tools for the measurement of structural racism in various populations (e.g., minoritized racial/ethnic groups, adolescents, pregnant populations).
- There is a lack of community input into developing social inequality metrics to ensure measures for different populations.
- There exists a lack of guidance for preterm birth researchers who have the expertise to model variables of structural racism (continuous, categorical, validated cut-point) and to identify confounders versus mediators or moderators (120).
- We see a lack of analytic approaches that can address the multiple layers of structural factors, individual factors, behavior, and biological changes that ultimately lead to a heterogeneous clinical outcome such as preterm birth.

Another consideration is how these exogenous factors affect the biological processes that drive PTB; these important connections are either neglected or inadequately studied. The latter issue is, in part, because researchers are often trained primarily within the silos of social determinants (SDOH) or biological determinants but not in the intersection of the two. Specifically, how can features of SDOH affect the immunological responses, and how might we measure and predict how these two broad areas interact to affect risk? One source of answers to this question is the development of biomarkers or predictive genetic factors that tie biology and social determinants together. For example, how do structural racism and social stressors modify the immune response that ultimately increases the risk of PTB, and how does genetic context modify this risk? Can we mediate only via biological intervention or determine which social factors most likely trigger biological responses and intervene there? Answers to these questions require filling the knowledge gaps within social and biological domains, but, more importantly, require defining measures that can establish key connections.

## Developing preventative treatments for PTB

Assessing treatment options for clinicians dealing with possible PTB are limited. Even presuming that an excellent measure or clinical sub-trait can predict risk and categorize high-risk pregnancies, a clinician’s dilemma is to provide proper care based on the specific indications provided by the marker (47, 48). However, at present, there are no appropriate and effective interventions or approved drugs to use during pregnancy that address specific risks. Hence, even if risk-induced, preterm labor-associated pathways were known, providers may be hesitant to determine risk because there are no available countermeasures to mitigate it. A clinically valuable biomarker may enable researchers to identify mechanisms by which the biomarker is produced and how it affects pregnancy outcomes; this knowledge can lead to conditions altering the risk relevant to specific pathways. Therefore, defining biomarkers may be of limited utility until we know how to use them to identify specific exposures and other pathogenic mechanisms.

Given a known and specific etiology, we must develop precision interventions, which have proven difficult for several reasons. Among the reasons is the failure to include pregnant women in many clinical trials, either for PTB or other conditions (Box 3). Although logical for the protection of the mother and fetus, this exclusion slows progress toward PTB treatments. Developing precision therapeutics has also proven difficult due to the lack of defined risk profiles specific to PTB. Without these definitions, neither biomarkers nor underlying etiologies can be assessed to determine the likelihood of a positive outcome. Even when treatments and management protocols exist, we have been relatively unsuccessful in determining who should get a particular intervention. For example, progesterone may be indicated in women with a short cervix, but this knowledge is the exception rather than the rule, as progesterone, in general, is of limited utility (49). Again, decomposing the heterogeneity of risk pathways will improve the likelihood of successful intervention and knowing specific biomarkers can be helpful in this endeavor.

BOX 3 Gaps in the development of pharmacotherapy for PTB

- Pregnant women are often excluded from clinical trials and translational studies in support of research in PTB outcomes.
- Most of the newer, innovative technologies have not been used in the PTB field to develop novel biomarkers and identify specific drug targets.
- Multi-disciplinary, collaborative approaches among obstetricians, neonatologists, pediatricians, and experts in AI, drug delivery, and drug development have rarely been established.
- Strict inclusion/exclusion criteria have not been applied to identify the appropriate target patient populations as defined by race/ethnic categories or risk biomarkers.
- Existing clinical measurements and biomarkers to differentiate patient phenotypes/risk profiles have not been fully leveraged in therapeutic development.
- Repurposing of most of the currently approved drugs has not been extensively explored for the potential for PTB treatment/prevention, especially the drugs that are currently used for other obstetrical disorders.

## Using biomarkers as proximal features to target prevention and treatment

### What is a biomarker?

Biomarkers can serve multiple needs for the research and clinical communities (50). The specific definition(s) and utilities of biomarkers are complex, and these useful tools can be characterized in many ways. A biomarker can be defined as “a…characteristic that is measured as an indicator of normal biological processes, pathogenic processes or responses to an exposure or intervention” (15). They serve as objective measures, usually based on lab analyses. The multiple purposes for biomarker development include diagnostics, monitoring, prognostics, prediction, pharmacodynamic/drug response, and safety. However, they can also define disease subtypes, as has been done for diabetes (51) and Alzheimer’s disease (52), and specify subtype-specific preventative and treatment options. Biomarkers can include several biological molecules or organisms, including changes in epigenetic marks, RNA expression, proteins, carbohydrates, lipids, and microbiome(s). They are dynamic features that can change in response to specific exposures, affecting health and disease; they can also reflect a specific genetic predisposition to respond in a particular way to each insult. Defining how biomarkers occur across the PTB syndrome can be critical in reducing risk.

**FIGURE 1:**
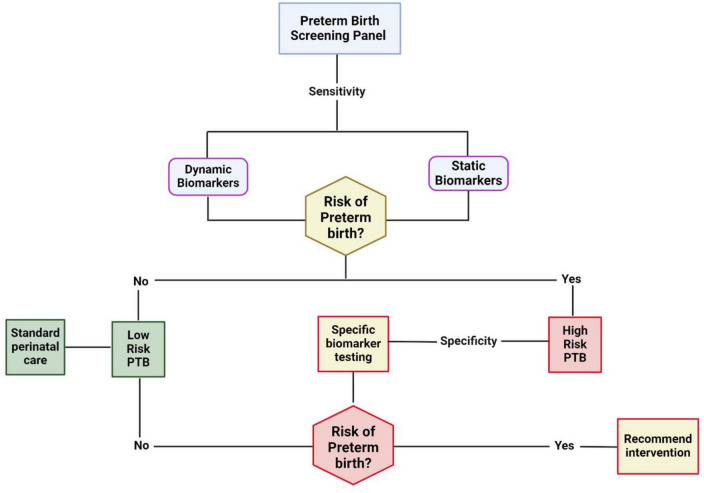
The necessity of biomarkers at various stages of gestation for risk classification, stratification, and management. Early risk can be assessed using highly sensitive static biomarkers like genetic markers, race/ethnicity, BMI, environment, behavior, etc. The dynamic markers are manifestations of an interaction between the pregnancy environment, maternal static factors, and other exposures during pregnancy. High-sensitivity biomarkers at this stage may stratify women at risk for preterm birth at low or high risk. A 2nd phase of classification can occur during various stages of pregnancy in the high-risk category. The specificity of biomarkers is critical to indicate specific system involvement (fetal vs. maternal), organ involvement (e.g., cervix, placenta, fetal membranes, decidua, myometrium), cell type involvement, and pathophysiologic mechanisms. The biomarkers are dynamic at this stage and likely involve multisystem, multi-organ, and multi biomarkers.

In contrast to the fluctuation or dynamic nature of biomarkers that respond to changes in physiology and the environment, genetic variation represents an essentially static, innate state that can also affect PTB risk (Figure 1). For example, it is estimated that the heritability of PTB is between 20% and 40% (53, 54), similar to the rates for hypertension and other complex conditions (55). However, in contrast to most other diseases, PTB arises through interactions between two organisms–the fetus and the mother- rather than from etiologies operating within a single patient. Possible interactions between these two are likely to be complex and critical to the syndrome (56). A key feature of genetics and biomarkers is that each can also be used to define phenotypic heterogeneity and, hence, underlying etiological diversity (57). In the above description of infection and inflammation, multiple factors can affect risk; the genetic risk will affect and even produce aberrant instances of those processes. Yet, a key feature of biomarker measurements vs. genetic factors that is essential to their utility is the timing of the measurement (58, 59). Although biomarkers may be associated with PTB, the same ones may not be associated with pregnancy or partuition (59). Knowing what, where, and when to measure them is important.

Biomarkers can additionally serve as readouts of specific exposures ranging from socioeconomic status factors, including features of the built environment, stress, explicit risk behavior such as smoking, physiological status derived from clinical traits such as high BMI, and even prior pregnancy history. A key aspect of these risk factors is that not all people exposed to them will deliver preterm. Related to this is whether most biomarkers are the cause or an effect of the PTB phenotypes. Current biomarkers being tested are likely downstream effect indicators and unlikely causal but this does not mean they are without utility. Causal factors may exist at the cell or nuclear level and not necessarily be reflected in the biological fluids generally used for biomarker testing. However, systemic factors that are entirely or partially at play in peripheral blood could also be a factor. Ultimately, with the combined knowledge from other research areas, such as those related to inflammation, it will eventually be possible to dissect the cause-and-effect relationship. This may be possible through specific analytical methods such as machine learning, mediation analyses, or, for genetic factors, Mendelian randomization (60).

Perhaps even more important is that single biomarkers are not likely to be highly predictive of PTB or personalized treatment outcomes. Hence, developing biomarker combinations or composites is likely the most useful approach, especially when using novel analytical methods such as machine learning, as has been done for cancer (61). Panels of biomarkers may also be useful in defining multiple and/or parallel pathways that underlie differing etiologies of PTB in different populations. Such biomarker combinations may relate to multiple measures over time instead of a single time point as predictors. For example, changes over time in a biomarker or combination of biomarkers may predict outcomes better than measures taken at one point in the pregnancy (62).

Once sufficient data are available, we can also ask whether the same biomarkers are important across populations. Populations can be defined in multiple ways, from genetic ancestries to women living in specific environments. A key feature of biomarker and genetic analyses is knowing when specific biomarkers should be used and for whom. We must approach the definition of biomarkers not as a one-size-fits-all but as one concerning the individuals being studied or treated.

To date, biomarkers for PTB include cervical fetal fibronectin (63), alpha-fetoprotein, C-reactive protein, and interleukin 6, but in most analyses, only single features are examined (63–66). These biomarkers are non-specific and do not generally indicate the involvement of a specific individual (mother or fetus) or organ system (maternal or fetal) (64, 67). The non-specificity of these biomarkers indicates overall system-level stress (at the single or multicellular level) or derangements in whole organs or within individuals (mother or fetus), and cannot determine the precise risk and etiopathology in discrete metabolic and signaling pathways. Multiple meta-analyses have shown the futility of these markers in predicting an impending outcome; however, Kacerovsky et al. and Cobo et al. have shown the utility of IL-6 and CRP in determining neonatal outcomes in preterm babies (68–70).

Several very recent systematic reviews (71–77) and some recent cohort studies have reported promising biomarker data to predict women at high risk for preterm labor (75, 78). New data on pregnancy-related phenotypes has presented some interesting possible multi-omic lines of investigation or combinations of multiple risk factors. A recent study on preeclampsia has indicated that cell free RNA (cfRNA) in maternal blood is predictive of outcome (79). A similar study on cfRNA showed an association with early and very early PTB (80). Another example is the prospective study of inflammatory marker ratios and post-partum depression (81). Advances in extracellular vesicle research and exosome-based biomarker studies have reported promising proteomic and miRNA markers in the exosomal lumen. Placental alkaline phosphatase-positive exosomes isolated from maternal plasma samples have provided valuable information regarding their utility in early diagnosis of high-risk status for spontaneous PTB (62, 82). Similarly, cervical length, microbiome profiles and other biologicals are also promising. (83–85) These studies can serve as models for future PTB studies.

### Where are we scientifically vs. clinically with biomarkers today?

To advance our attempts to understand PTB there is a need for a systems biological approach incorporating multi-omics data and artificial intelligence/machine learning to manage the data appropriately (45). The latest attempts to determine fetal-specific biomarkers in extracellular vesicles at various stages of pregnancy from minimally invasive samples (i.e., maternal blood) are promising (62, 82, 86).

The Preterm Birth International Collaborative (PREBIC) has conducted multiple systematic reviews to identify the knowledge gaps in biomarker discovery, development, and testing to predict high-risk status (45, 87–90). The Global Alliance to Prevent Prematurity and Stillbirth (GAPPS) has also taken on this topic (91, 92) The PREBIC systematic reviews assessed literature published since the ‘70s and covered various topics. These reviews were divided into various aspects of biomarkers of preterm birth primarily based on how they are selected, used for trials, and by their ability to predict a high-risk status for stratifying mothers at risk for preterm birth early in pregnancy. The biomarkers discovered and tested were based on a wide variety of samples, including, but not limited to, maternal plasma, serum, urine, cord plasma/serum, amniotic fluid, exosomes, and cervicovaginal fluids (45, 93). The reviews were based on how markers were used in identifying risk: (1) single biomarkers, primarily detected using sandwich ELISA or radioimmunoassay approaches (89); (2) multiple biomarkers tested using multiplexing of reagents using platforms such as Luminex or flow cytometry (87); (3) multiple markers identified using proteomics data (88), (4) genetic variations and associated risk (94, 95); and, (5) using the vaginal microbiome as biomarkers (90). The reviews encompassed many biomarkers being tested in both fetal and maternal compartments. Various biomarker production sites (tissues or cells of origin) and functional roles in diverse pathophysiological processes associated with preterm labor have been identified and reported.

Data extracted from the PREBIC reviews could not be used to conduct meta-analyses for two reasons. First, there was a lack of common factors and study design identified that applied to most of the studies, and second, there were elements that were unique to the specific approaches used for identifying biomarkers. Because of the syndromic nature of the disease and current gaps in our knowledge, biomarker discovery for PTB is more complex than discovery for other phenotypes and disease states. Pregnancy is a state where two distinct physiologies and immunological (paternal antigens) systems share an environment for 9 months or until the fetus is mature and ready to separate from the mother. Pathophysiologies may arise on the maternal side (mother as a patient) or the fetal side (fetus as a patient). Therefore, the culmination of pregnancy must be a coordinated event between the two physiologies. Still, complicated pregnancies may exhibit pathophysiologic pathways initiated on the maternal or fetal sides but can ultimately involve both. The kinetics of biomarker generation by the “patient” indicative of an underlying risk is critical to determine the organ involvement, the biomarker to be evaluated, and the type and accessibility of samples to be collected. Many studies have tried to retrospectively identify risk exposures after identifying a biomarker’s difference in expression between cases and controls (96, 97). However, we argue most biomarker panels are not precise in determining various exposure risks other than indicating an overall disturbance, but not where or when. Therefore, the following gaps must be addressed to be able to implement biomarkers effectively:

1. Lack of knowledge of organs at risk and biomarkers expected to be derived from each organ–during pregnancy, both maternal and fetal organs can become dysfunctional and generate pathways inducing preterm labor (98). Although this knowledge is difficult to acquire before running a panel of biomarkers, an organ system’s involvement based on potential risk exposure will help to determine (1) the type of sample needed for measurement, (2) the timing of sample collection, and (3) the biochemical nature of the biomarker.
2. Population diversity is generally not accounted for–as stated above, genetic determinants of preterm birth risks and population diversity are not often well reported. The variation that environment or group diversity can produce in clinical and biomarker manifestation has not been considered, especially in studies done between the ‘80s and early 2000s.

The above factors contribute to heterogeneities that prevent meta-analyses of data from determining whether the reported biomarkers are reproducible in multiple settings. Other issues have been identified in biomarker research. These are detailed in the systematic reviews cited above but summarized here.

1. Type of sample, timing of collection, processing, and storage conditions.
2. Retrospective samples, selection of biomarkers, and rationale for selection.
3. Sample size, assay type, and analytical approaches.
4. Validation and replication of data.
5. Interpretation and reporting.

Heterogeneities created by all of these factors can prevent useful biomarker identification.

Recently, biomarker studies have been more robust and have addressed many of the abovementioned concerns (99) that have hampered biomarker discovery and development in the past. For example, a PREBIC biomarker working group review recently reported concerted efforts by investigators to identify biomarkers, most of which were agnostic. A review by Lamont et al. (45) concluded that the combination of biophysical, biochemical (100), immunological (101), microbiological (33, 102, 103), fetal cell (86), exosomal (82, 104, 105), or cell-free RNA (106) at different gestational ages, integrated as part of a multivariable predictor model or machine learning approaches (45), may be necessary. Fetal-specific tags are used for this purpose, and reports indicate the determination of the high-risk status of spontaneous preterm birth as early as the first trimester of pregnancy (62, 82, 104, 107). Although many of these studies are promising, they must be replicated using prospectively analyzed samples in larger and more diverse cohorts.

### Where to begin discovering, testing, and replicating biomarkers

**FIGURE 2:**
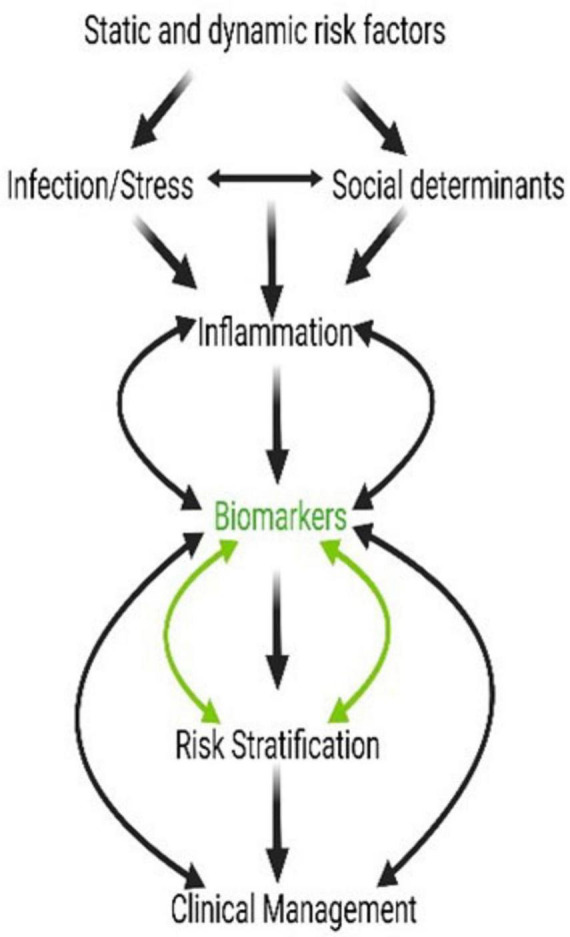
Social stressors, inflammation, and intervention can all be linked via biomarkers. Biomarkers of pregnancy-associated risk and genetic, biomolecular, and biochemical changes at the cellular level should result in dynamic and measurable level changes. These biomarkers are risk indicators and diagnostic markers of pathology and could also be used as indicators of response and prognosis after an intervention.

Risk factors can be static (e.g., genetic, socioeconomic, BMI) or dynamic (manifestations of interactions between static factors and the pregnancy environment or exposures during pregnancy). As shown in Figure 2, an infection developing along with social risk factors (e.g., access to care, financial concerns, and familial pressures) and psychological stress arising during pregnancy can generate an inflammatory environment. Inflammation is a common feature of PTB, irrespective of an infectious etiology, and can arise from a sterile risk development process. However, these inflammatory environments are often self-contained by both fetal and maternal compartments, do not impact pregnancy, and do not result in adverse events. In contrast, when the inflammatory response (fetal and/or maternal) becomes overwhelming, specifically the fetal inflammatory response triggering a shift in the balance of quiescent organs, their manifestation in different intrauterine organ systems can produce varying pathologies (2). The biomarkers from these pathologies, whether the fetal membranes, placenta, cervix, decidua, or myometrium, are indicative of underlying risk at a specific affected organ system, can be used for risk and treatment stratification (98). Currently, no biomarkers are available to determine the exposure risk, organ(s) impacted, and the risk propagation to different regions of the uterine compartments. Our current approaches attempt to determine a universal biomarker or a set of biomarkers indicative of an overall disturbance. This approach is likely insufficient to predict and treat a complex set of phenotypes like PTB. Dynamic biomarkers indicative of exposure, development of pathologies, propagation of pathologic mechanisms, and their manifestations should be employed and monitored for proper management.

Appropriately defining biomarkers is cumbersome as we are still trying to define various organ systems, their involvement in the PTB process, and how they differ from changes during normal-term pregnancy. Based on our existing knowledge of pathophysiologic mechanisms of preterm labor pathway development, we postulate that machine learning approaches can be developed to enhance prediction capabilities at different stages (99). However, this will require much more data from different sources and scientific, sociological, and clinical disciplines than is currently available for analysis.

We will need to tie our research to standard clinical management to choose biomarkers to be assessed in a range of disease development stages, and not necessarily follow the same biomarkers throughout pregnancy. We propose that the connectors between scientific domains described above may best be determined by developing PTB-related biomarkers at different stages of pregnancy. For example, one biomarker detected based on inflammation at an early gestational age may promote an endocrine change later in pregnancy in women at particular risk. Similarly, there may be women who are highly susceptible or resilient to PTB based on innate biological features or genetic predispositions. We propose that we can use these factors to define how to intervene based on a combination of genetic and exogenous factors simultaneously. Here, we define biomarkers with the understanding that they are not necessarily causative but rather readouts of other biological and social factors that affect PTB risk. Determining the mechanism and causation will be necessary, but we are not yet there.

## Filling in the gaps

In this article, we identified gaps in three critical areas of preterm birth-related risk and management to illustrate that much more knowledge must be gained before truly addressing generally useful biomarker discovery and testing. The critical gaps in the three main domains are listed in Boxes 1–3. However, we are not recommending waiting until we fill all knowledge gaps; rather, we propose that as we develop more knowledge within the domains, we will need to assess the biomarkers associated with each feature and, more importantly, how they relate to each other. We need to define biomarkers that can be used to connect domains and therefore define risk better than we have historically, using a single research or clinical domain. For example, some biomarkers of inflammation operate independently of SDOH, and some SDOH features operate independently of inflammation, but some will likely be interrelated.

**FIGURE 3:**
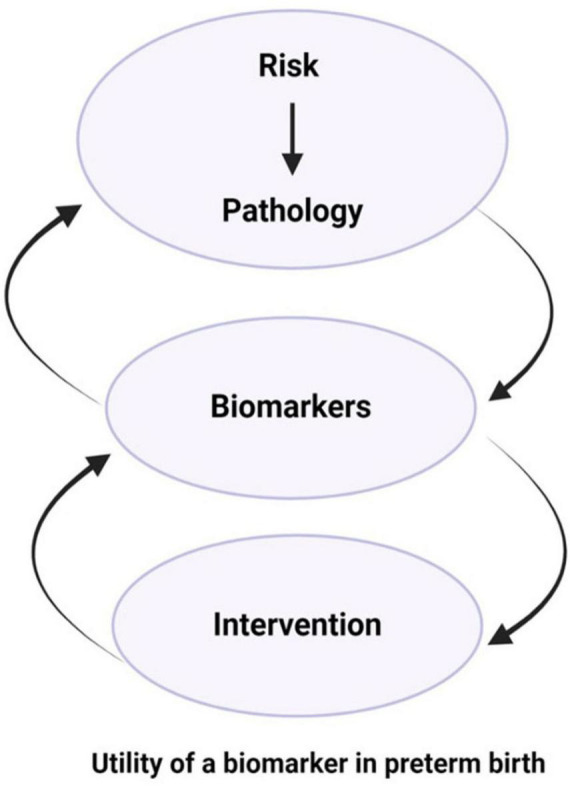
Biomarkers as indicators of risk-induced pathophysiologic mechanisms, progression of disease, and prognosis of interventions. Biomarkers can be employed to identify risk and determine targets for intervention, whether therapeutic targets or tools for monitoring response to interventions.

Additionally, we need to recognize that the most at-risk populations are in low and middle income countries (LMIC) and often marginalized populations in high income countries. Therefore, the development of useful biomarkers needs to consider cost and utility in these environments, including the development of low cost platforms that can be harmonized. The goal is to make biomarker panels that are highly predictive and useful, universally accessible. Cognizance of cost and utility is essential given the global distribution of PTB. Finally, we again emphasize the need to recognize that a particular biomarker may not predict the same outcome across multiple heterogeneous measures. Therefore, validation will remain a challenge, but one that can be overcome with sufficient effort. Finding the appropriate interactions can fill in the gaps between domains 1 and 2 (Figures 1, 3) and help us assess the most appropriate interventions.

## Summary

1. Biomarkers can be risk exposure indicators and lead to the discovery of pathology.
2. Biomarkers can be indicators of organ involvement.
3. Biomarkers can identify targets for interventions (molecules, cells, organs).
4. Biomarkers can provide management guidelines.
5. Biomarkers can determine the prognosis of an intervention.
6. Biomarkers may differ by study population or previous exposures, and this heterogeneity needs to be explicitly considered.

## PREBIC North America Workshop Participants

**Infection/Inflammation**

Addy Cecilia Helguera-Repetto, Departamento de Inmunobioquímica, Instituto Nacional de Perinatología (INPer), Mexico City, Mexico, Alison Eastman, Department of Obstetrics and Gynecology, Vanderbilt University Medical Center, Nashville, TN, United States, Angela M. DeTomaso, Department of Pathology, Case Western Reserve University, Cleveland, OH, United States, Hana Totary Jain, Department of Molecular Pharmacology and Physiology, University of South Florida, Tampa, FL, United States, Kristen N. Noble, Department of Pediatrics, Vanderbilt University Medical Center, Nashville, TN, United States, Orlando Cervantes, Department of Global Health, University of Washington, Seattle, WA, United States, Stephen A. McCartney, Department of Obstetrics and Gynecology, University of Washington, Seattle, WA, United States, Veronica Zaga-Clavelina, Departamento de Inmuno-Bioquímica, Instituto Nacional de Perinatología, Ciudad de México, Mexico.

**Social Determinants of Health**

Ashley Hill, Division of Community Health Sciences, University of Illinois Chicago, Chicago, IL, United States, Corrie Miller, Department of Obstetrics and Gynecology, University of Hawaii, Honolulu, HI, United States, Elizabeth Bonney, Department of Obstetrics, Gynecology and Reproductive Sciences, University of Vermont College of Medicine, Burlington, VT, United States, Felipe Vadillo-Ortega, Jefe de la Unidad de Vinculación de la Facultad de Medicina, Instituto Nacional de Medicina Genómica, Universidad Nacional Autónoma de México, Mexico City, Mexico, Jadia Mi, Department of Women and Children’s Health, Kings College London, London, United Kingdom, Natacha De Genna, Department of Psychiatry, University of Pittsburgh, Pittsburgh, PA, United States, Sarah England, Department of Obstetrics and Gynecology, Washington University School of Medicine, St. Louis, MO, United States, Sarah Vaughan, Department of Public Health, Wayne State University, Detroit, MI, United States, Sarka Lisonkova, Department of Obstetrics and Gynecology, University of British Columbia, Vancouver, BC, Canada.

**Translational Research/Therapeutics**

Brice Gaudilliere, Department of Anesthesia, Stanford University School of Medicine, Stanford, CA, United States, Carlos Hernán Becerra Mojica, Gynecology and Obstetrics, Universidad Industrial de Santander, Bucaramanga, Santander, Colombia, Egle Bytautiene Prewit, Department of Obstetrics and Gynecology, The University of Texas Health, San Antonio, TX, United States, Emily Hamburg-Shields, Department of Reproductive Biology, School of Medicine, Case Western Reserve University, Cleveland, OH, United States, Erik Rytting, Department of Obstetrics and Gynecology, The University of Texas Medical Branch, Galveston, TX, United States, Gygeria Manuel, Obstetrics and Gynecology, University of Washington, Seattle, WA, United States, Marian Kacerovsky, Department of Obstetrics and Gynecology, University Hospital Hradec Kralove, Charles University, Hradec Kralove, Czechia, Mehmet R. Genc, Department of Obstetrics and Gynecology, University of Florida College of Medicine, Gainesville, FL, United States, Sam Mesiano, Department of Reproductive Biology, School of Medicine, Case Western Reserve University, Cleveland, OH, United States, Stephen J. Lye, Lunenfeld-Tanenbaum Research Institute, Mount Sinai Hospital, Toronto, ON, Canada.

**Genetics/Biomarkers**

Alejandra Rondon Torres, Instituto Nacional de Cardiologia, Ciudad de México, Mexico, Arturo Flores-Pliego, Instituto Nacional de Perinatología Isidro Espinosa de los Reyes, Mexico City, Mexico, Daniel Eduardo Sandoval Collin, Instituto Nacional de Medicina Genómica: Ciudad de Mexico, Ciudad de Mexico, Mexico, Daniel Gonzalez, Department of Clinical Laboratory Sciences, University of Texas Medical Branch at Galveston, Galveston, TX, United States, Fewa; Fewa; Laleye, Women and Children’s Health, King’s College London, London, United Kingdom.
